# Live Biosensors for Ultrahigh-Throughput Screening of Antimicrobial Activity against Gram-Negative Bacteria

**DOI:** 10.3390/antibiotics10101161

**Published:** 2021-09-24

**Authors:** Margarita N. Baranova, Polina A. Babikova, Arsen M. Kudzhaev, Yuliana A. Mokrushina, Olga A. Belozerova, Maxim A. Yunin, Sergey Kovalchuk, Alexander G. Gabibov, Ivan V. Smirnov, Stanislav S. Terekhov

**Affiliations:** 1 Shemyakin-Ovchinnikov Institute of Bioorganic Chemistry of the Russian Academy of Sciences, 117997 Moscow, Russia; baranova@ibch.ru (M.N.B.); babikova.pa@phystech.edu (P.A.B.); kudzhaev_arsen@mail.ru (A.M.K.); yuliana256@mail.ru (Y.A.M.); o.belozyorova@gmail.com (O.A.B.); yuninma@gmail.com (M.A.Y.); xerx222@gmail.com (S.K.); 2 Department of Chemistry, Lomonosov Moscow State University, 119991 Moscow, Russia

**Keywords:** ultrahigh-throughput screening, live biosensors, antibiotic discovery, gram-negative pathogens, microfluidic droplet cocultivation, efficient promoters, polymyxins, colistin biosynthetic gene cluster, single cell, multi-omics

## Abstract

Gram-negative pathogens represent an urgent threat due to their intrinsic and acquired antibiotic resistance. Many recent drug candidates display prominent antimicrobial activity against Gram-positive bacteria being inefficient against Gram-negative pathogens. Ultrahigh-throughput, microfluidics-based screening techniques represent a new paradigm for deep profiling of antibacterial activity and antibiotic discovery. A key stage of this technology is based on single-cell cocultivation of microbiome biodiversity together with reporter fluorescent pathogen in emulsion, followed by the selection of reporter-free droplets using fluorescence-activated cell sorting. Here, a panel of reporter strains of Gram-negative bacteria *Escherichia coli* was developed to provide live biosensors for precise monitoring of antimicrobial activity. We optimized cell morphology, fluorescent protein, and selected the most efficient promoters for stable, homogeneous, high-level production of green fluorescent protein (GFP) in *E. coli*. Two alternative strategies based on highly efficient constitutive promoter pJ23119 or T7 promoter leakage enabled sensitive fluorescent detection of bacterial growth and killing. The developed live biosensors were applied for isolating potent *E. coli*-killing *Paenibacillus polymyxa* P4 strain by the ultrahigh-throughput screening of soil microbiome. The multi-omics approach revealed antibiotic colistin (polymyxin E) and its biosynthetic gene cluster, mediating antibiotic activity. Live biosensors may be efficiently implemented for antibiotic/probiotic discovery, environmental monitoring, and synthetic biology.

## 1. Introduction

Global use of antimicrobials provokes intensive antimicrobial resistance (AMR) selection. AMR represents a threat to sustainable development, leading to 11 million deaths annually [1,2]. The recent COVID-19 pandemic resulted in a significant increase in antibiotic sales [3] and extensive antibiotic use without proper clinical indication [4]. Hence, the overuse of antibiotics lays the foundations for further resistome propagation and multi-resistance evolution. Further deterioration in this field threatens the emergence of epidemics caused by multiresistant pathogens and their subsequent persistence in the population under selection pressure.

Gram-negative pathogens represent a particularly dangerous cohort, including three of five urgent threats highlighted by the Centers for Disease Control and Prevention (CDC) [5]. New antibiotics targeting resistant Gram-negatives have been approved, but most of them belong to existing classes of antibiotics, and resistance to them has already emerged [6]. Gram-negative bacteria have an outer membrane, a protective and unique feature that distinguishes them from Gram-positive bacteria. This shield provides an efficient barrier for a vast variety of antimicrobials. Together with acquired resistance mechanisms, like mutations in chromosomal genes or mobile genetic elements carrying resistance genes, this provides a challenge to medication, often unresolvable [7].

Despite the urgent antibiotic rediscovery problem [8], classical antibiotic-producing species still provide a source for new drug candidates [9]. However, exotic microbial communities represent a more promising reservoir for the isolation of new antibiotics [10]. Recently, we developed an ultrahigh-throughput microfluidic platform for biodiversity screening [11]. This technology is based on single-cell cultivation of microorganisms in isolated microcompartments of double water-in-oil-in-water emulsion with subsequent isolating phenotypes of interest by fluorescence-activated cell sorting (FACS). More than 10,000 single bacterial clones may be screened for antibiotic activity in a second to isolate the most efficient antibiotic producers [12] or resistant strains [13]. This productivity enables deep functional profiling of microbiota communities [12] and the discovery of new molecular mechanisms of resistance [14]. The critical step of this technique is a coencapsulation of a highly fluorescent reporter GFP-producing bacterial strain together with single cells from the microbiome followed by their cocultivation in droplet compartments. Efficient bacterial killers are subsequently selected with FACS by a low GFP fluorescence level in a minor subpopulation of droplets. Previously, this platform was implemented for deep profiling of anti-*S. aureus* activity [11,12,13]. In this study, cell morphology, fluorescent protein nature, and promoter efficiency were optimized to adopt this strategy for extensive anti-Gram-negative screening based on model bacteria *E. coli*. Common laboratory *E. coli* strains, including Rosetta, BL21(DE3), TG1, XL-1, and SHuffle T7, were investigated to maximize GFP fluorescence and homogeneity. Two different green fluorescent proteins, i.e., TagGFP2 [15] and sfGFP [16], were examined as reporters in live biosensors. Moreover, the efficiency of GFP production under the control of different promoters was compared, including highly efficient constitutive *E. coli* promoters, i.e., pEm7 [17], pglpT [18], pJ23119 [19], OXB20 [20], and leaking T7 promoter.

We obtained that both highly efficient constitutive promoter pJ23119 and T7 promoter leakage enable sensitive fluorescent detection of bacterial growth and killing. Live biosensor based on BL21(DE3) strain, producing sfGFP via leaking T7 promoter, outperformed pJ23119 in terms of fluorescence level, while the fluorescence distribution of T7-based reporters was higher than pJ23119. Finally, pJ23119-sfGFP BL21(DE3) reporter cells were applied for a proof-of-concept soil microbiome screening. In a single round of screening, a potent *E. coli*-killing *Paenibacillus polymyxa* P4 strain was isolated and analyzed by complex multi-omics strategy, including activity-based metabolomics and genomics. Colistin (polymyxin E) was determined as a key metabolite mediating anti-Gram-negative antibiotic activity. The identified biosynthetic gene cluster (BGC) of colistin displayed close similarity to BGCs of polymyxin A [21], D-Dab_3_-polymyxin B [22], and polymyxin E [23]. However, it did not contain the epimerization domain in module 3, unlike all previously published BGCs of polymyxins. We believe that the developed live biosensors may be efficiently implemented for ultrahigh-throughput screening of antimicrobial activity against gram-negative bacteria for antibiotic/probiotic discovery, environmental monitoring, and synthetic biology.

## 2. Results

### 2.1. General Requirements for Live Biosensors Applied in Ultrahigh-Throughput Screening

**Figure 1:**
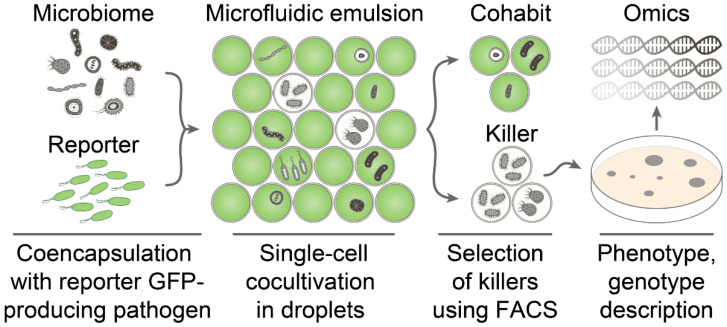
The general pipeline of ultrahigh-throughput screening of antimicrobial activity. The bacterial community is encapsulated with reporter GFP-producing pathogen in biocompatible droplets of microfluidic double water-in-oil-in-water emulsion. Cocultivation of bacterial community with reporter bacteria in droplets results in two distinct populations containing bacterial cohabits and killers. The latter is selected by a low level of GFP fluorescence by FACS. The selected droplets are plated on agar to regenerate culturable killers analyzed by activity-guided metabolomics and genomics. Detailed phenotype and genotype description enable identifying antibiotics and their biosynthetic gene clusters.

Ultrahigh-throughput screening of antimicrobial activity is based on a single-cell encapsulation of microbiome representatives together with fluorescent protein-producing reporter bacteria followed by isolation of droplets with inhibited growth of reporter bacteria using FACS (Figure 1).

The critical component of this platform is a reporter pathogen strain. It must follow certain criteria, essential for efficient screening: (1) Cells must have regular morphology; aggregates or big non-uniform cells are undesirable; (2) production of fluorescent protein should be constitutive and homogeneous in population; and (3) a high level of cell culture fluorescence is required for precise detection of antimicrobial activity. A model Gram-negative bacteria *E. coli* was optimized following these criteria to provide efficient, live biosensors for ultrahigh-throughput screening and sensitive antimicrobial activity detection.

### 2.2. Selection of GFP-Producing Strain

**Figure 2:**
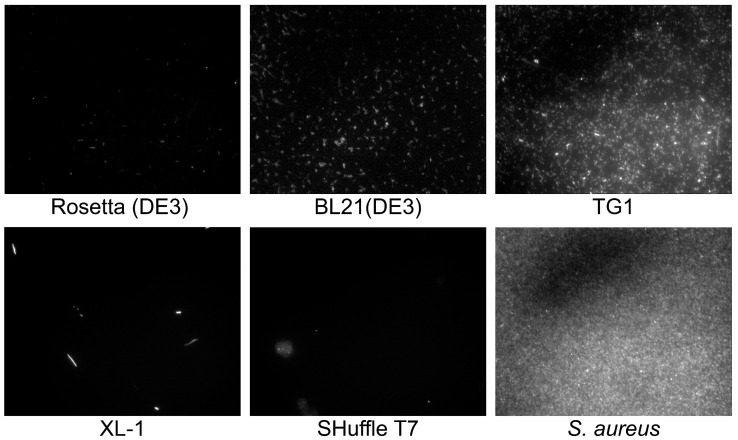
Cell morphology of different *E. coli* strains visualized by fluorescence microscopy. GFP-producing reporter *S. aureus* cells were used as a control.

Cell morphology plays an important role for reporter strain selection since cell aggregate clot microfluidic channels, while big and irregular cells tend to sediment in fluidics. Moreover, cell aggregates and non-uniformity may influence regular droplet occupancy. Hence, common laboratory *E. coli* strains including Rosetta (DE3), BL21(DE3), TG1, XL-1, and SHuffle T7 were transformed with pYTK047 plasmid for constitutive production of GFP [24], and cell morphology was visualized by fluorescence microscopy (Figure 2).

BL21(DE3) and TG1 strains of *E. coli* were suitable as a template for the creation of live biosensors since they provided homogeneous cell cultures with a high level of cell fluorescence. Other strains were inappropriate as a reporter. Rosetta (DE3) had a mediocre fluorescence. Xl-1 had a stretched morphology with a high number of odd rod cells. SHuffle T7 formed cell aggregates. Further, *E. coli* BL21(DE3) was used for live biosensors’ engineering.

### 2.3. Stable and Homogeneous Production of GFP in E. coli

**Figure 3:**
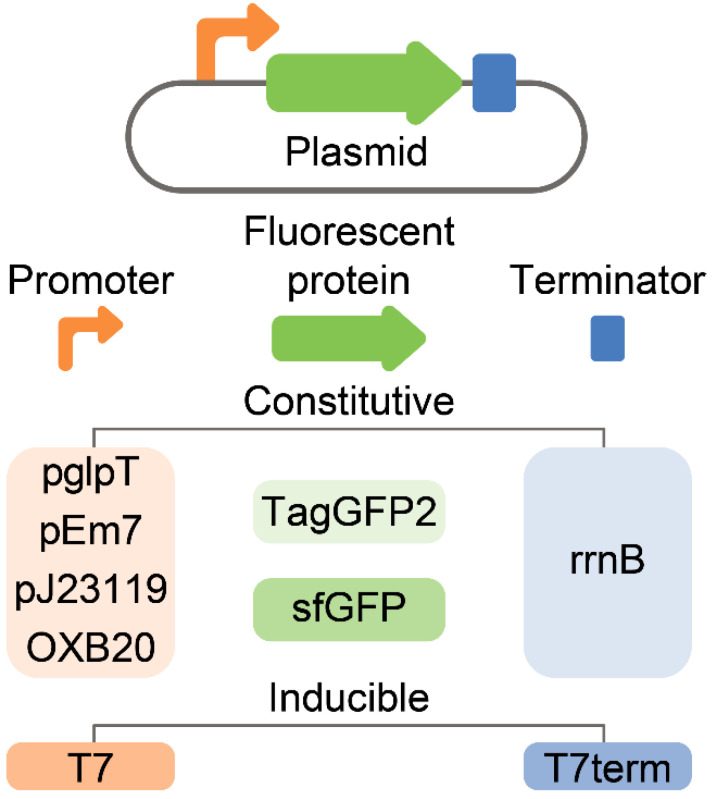
A panel of plasmids that were used for the optimization of *E. coli*-based live biosensors. All genetic constructs were constructed on the same high-copy number template vector. Two different fluorescent proteins, i.e., TagGFP2 and sfGFP, were used. Constitutive (pglpT, pEm7, pj23119, and OXB20) and inducible (T7) promoters were compared in terms of efficiency, homogeneity of cell fluorescence, and stability.

Different fluorescent proteins and promoters were compared to maximize the fluorescence of live *E. coli* culture (Figure 3).

**Figure 4:**
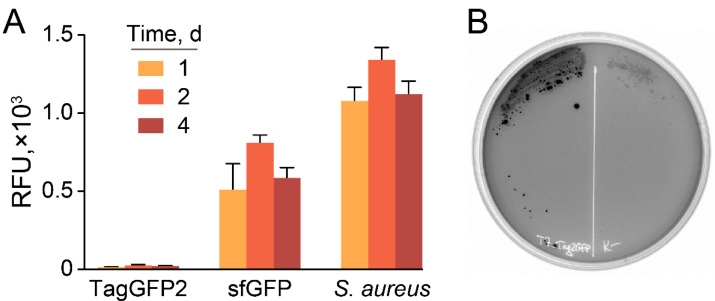
Hyperproduction of TagGFP2 fluorescent protein results in plasmid loss. (**A**) Fluorescence of cell cultures transformed with synonymous plasmids encoding TagGFP2 and sfGFP fluorescent proteins under the control of T7 promoter, induced by the addition of 1 mM IPTG. GFP-producing reporter *S. aureus* cells were used as a control. Data represent the mean of three biological replicates ± SD. (**B**) A representative plate with *E. coli*-producing TagGFP2 under control of T7 promoter, induced by 1 mM IPTG.

While TagGFP2 was declared non-toxic to the host [15], we observed dramatically decreased fluorescence of cell cultures in *E. coli* transformed with plasmids under control of T7 promoter compared with the same constructs coding sfGFP fluorescent protein (Figure 4A).

Moreover, TagGFP2-producing plasmids based on T7 promoter tend to be lost under induction, resulting in a highly heterogeneous population with varying fluorescence of colonies (Figure 4B). Hence, sfGFP was used as a reporter fluorescent protein.

**Figure 5:**
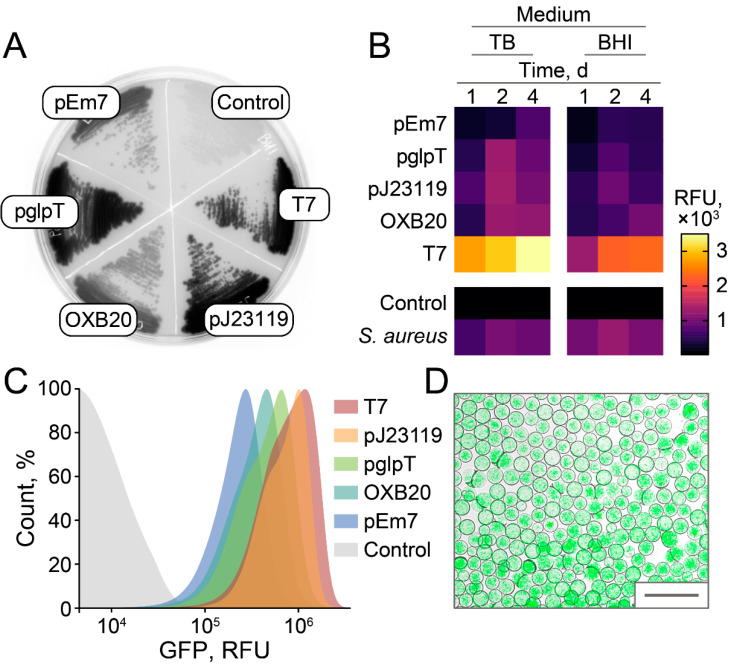
Efficacy of different promoters for constitutive and homogeneous production of GFP in *E. coli*. (**A**) A representative BHI-agar plate with *E. coli,* producing sfGFP under control of various promoters. Constitutive *E. coli* promoters, i.e., pEm7, pglpT, pJ23119, and OXB20, were compared with leaking T7. *E. coli* cells transformed with empty vector without sfGFP insert were used as a negative control. (**B**) Fluorescence of bacterial cultures obtained after 1, 2, and 4 days of cultivation in TB or BHI medium. Relative fluorescence units (RFU) are presented as a heatmap indicating bulk fluorescence of cell cultures. GFP-producing reporter *S. aureus* cells were used as a positive control. Data represent the mean of three biological replicates. (**C**) Flow cytometry of bacterial cultures producing sfGFP obtained after 2 days of cultivation in BHI medium. (**D**) Live biosensors producing sfGFP under control of pJ23119 promoter after 1 day of cultivation in droplets of microfluidic double water-in-oil-in-water emulsion. Scale bar: 100 μm.

The efficiencies of different promoters were compared to maximize GFP fluorescence in *E. coli*. Brain Heart Infusion (BHI) was used as a basal medium since it enables culturing a variety of microorganisms, which is preferred for subsequent ultrahigh-throughput screening of antimicrobial activity. Surprisingly, efficient T7 leakage was observed in *E. coli* BL21(DE3) (Figure 5A). Highly efficient constitutive *E. coli* promoters, i.e., pEm7, pglpT, pJ23119, and OXB20, were compared with leaking T7 in agar plates (Figure 5A), culture medium (Figure 5B), and flow cytometry (Figure 5C). Leaking T7 results in 2.3–8.9 times higher fluorescence of bacterial cultures in comparison with constitutive *E. coli* promoters. The pJ23119 was the strongest constitutive promoter that enabled stable and homogeneous fluorescence of cell cultures, similar to GFP-producing reporter *S. aureus* cells used previously (Figure 5B). While live biosensors based on leaking T7 promoter outperformed pJ23119 in cell cultures, the cell fluorescence distribution of T7-based reporters was higher (Figure 5C). The fluorescence levels of cell cultures were 1.3–1.6 times higher with the TB medium. However, we suggest that BHI is more preferred to maintain broad biodiversity in emulsion culture.

Hence, we propose that alternative strategies based on highly efficient constitutive promoter pJ23119 or T7 promoter leakage enabled sensitive fluorescent detection of bacterial growth and killing. Live biosensors producing sfGFP under control of pJ23119 promoter were encapsulated in biocompatible droplets of microfluidic double water-in-oil-in-water emulsion with occupancy of ~5 *E. coli* cells per droplet. Cultivation of reporters in emulsion resulted in efficient bacterial growth, providing highly fluorescent droplet compartments (Figure 5D) suitable for ultrahigh-throughput screening of antimicrobial activity.

### 2.4. Ultrahigh-Throughput Screening of Antimicrobial Activity

**Figure 6:**
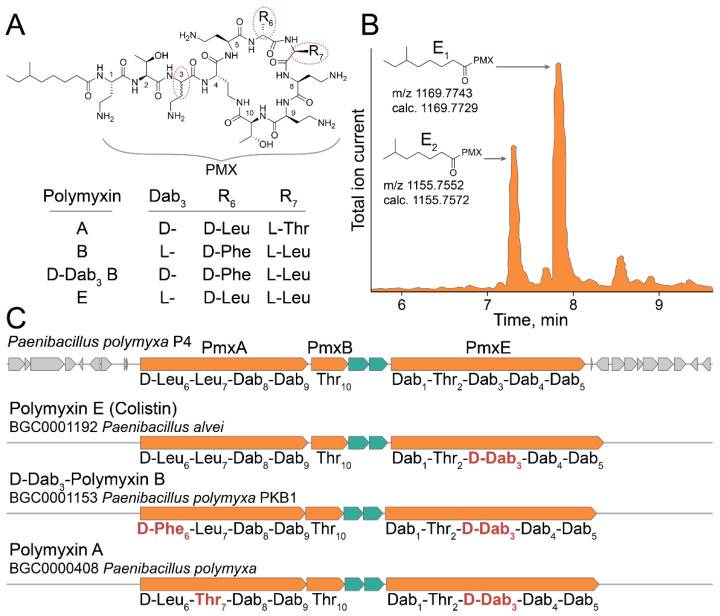
Multi-omic characterization of *Paenibacillus polymyxa* P4 strain selected by ultrahigh-throughput screening of anti-Gram-negative antibiotic activity using the developed live biosensor. (**A**) Polymyxin E (colistin) was identified as a major secondary metabolite of *P. polymyxa* P4 active against Gram-negative bacteria. The chemical structures of polymyxin E and related polymyxins A, B, and D-Dab_3_ B produced by distinct *Paenibacillus* are presented. PMX—colistin peptide backbone. (**B**) LC-MS chromatogram of active fraction of *P. polymyxa* P4 metabolites. Specific peaks of polymyxin E_1_ and polymyxin E_2_ are presented with their experimental and calculated [M+H]^+^ molecular ion masses. (**C**) BGC of polymyxin E identified in the genome of *P. polymyxa* P4. Core NRPSs (PmxA, PmxB, and PmxE) and ABC transporters are colored with orange and aquamarine, respectively. Predicted amino acid specificity of NRPS modules are presented. Related BGCs of polymyxin A [21], D-Dab_3_-polymyxin B [22], and polymyxin E [23] are presented with their predicted modular specificities. Distinct modular specificities are highlighted with red.

A model ultrahigh-throughput screening of antimicrobial activity was performed to demonstrate the efficiency of the developed live biosensors. Soil microbiome isolated in the Moscow region was used as a source of bacterial biodiversity. GFP-producing live biosensors were coencapsulated with ~10^6^ soil microbiome bacteria, followed by cocultivation, selection, and regeneration of culturable anti-*E. coli* bacteria. Potent *E. coli*-killing *Paenibacillus polymyxa* P4 strain was isolated in one round of selection. Metabolomic analysis revealed that polymyxin E (colistin) is the major secondary metabolite active against Gram-negative bacteria. Polymyxins represent closely related lipopeptide antibiotics having a high number of positively charged 2,4-diaminobutyric acid (Dab) residues and hydrophobic residues of Leu and Phe (Figure 6A). Similar to previously identified *Paenibacillus alvei* B-LR [23], *P. polymyxa* P4 produced two analogous polymyxins E_1_ and E_2_ that differ by 6-methyloctanoic acid and 6-methylheptanoic acid moieties, respectively (Figure 6B).

Whole genome sequencing of *P. polymyxa* P4 enabled identifying the biosynthetic gene cluster (BGC) of polymyxin E (Figure 6C). *P. polymyxa* P4 polymyxin E BGC has the same architecture as all polymyxin BGCs mediating the production of polymyxin A [21], D-Dab_3_-polymyxin B [22], and polymyxin E [23] identified previously. Polymyxin E BGC encodes d-Leu instead of d-Phe in module 6 of D-Dab_3_-polymyxin B, Leu instead of Thr in module 7 of polymyxin A, and Dab instead of D-Dab in module 3 of all known polymyxins. While modules 6 and 7 encode amino acid residues varying between polymyxins, module 3 does not have an epimerization domain. Hence, we predicted that the identified polymyxin E produced by *P. polymyxa* P4 has natural L-stereochemistry. It was recently shown that the epimerization domain of module 3 may be functional, at least in the case of *P. polymyxa* PKB1 strain producing D-Dab_3_-polymyxin B [22]. Therefore, the stereochemistry of Dab_3_ is questionable for some polymyxins. However, it is unambiguous for the identified polymyxin E produced by *P. polymyxa* P4.

## 3. Discussion

Estimates indicate that antibiotics not yet discovered are likely to be produced at frequencies as low as ≤1 in 10^7^ in fermentation broths from random actinomycetes [25]. Hence, deep profiling of antimicrobial activity of microbiomes on a single-cell level provides a new perspective to antibiotic discovery. The miniaturization of antibiotic activity assays is an essential step forward to the next-generation screening platforms. Microfluidic technologies enable single-cell bacterial culturing [26], enzymatic activity screening [27], and antimicrobial activity profiling [28]. Biocompatible droplet microcompartments allow transitioning from classical 2D culture on the surface of agar plates to 3D emulsion culture. This transition results in a dramatic increase in explored diversity since ~10^6^–10^9^ unique bacterial clones may be cultured in 1 mL of 100–10 µm emulsion droplets instead of ~10^2^–10^3^ clones on a single agar plate.

Here, we described how ultrahigh-throughput technologies could be applied for antimicrobial activity screening against Gram-negative bacteria. Live biosensors based on engineered GFP-producing *E. coli* enable detecting bacterial antagonism of individual bacterial clones cultured in droplet microcompartments. Different *E. coli* strains, GFPs, and promoters were tested to optimize cell morphology and maximize cell fluorescence. T7 promoter leakage enables sensitive fluorescent detection of bacterial growth and killing regardless of IPTG or lactose induction using common laboratory strain BL21(DE3) and high-copy number plasmid. Highly efficient constitutive promoter pJ23119 may be an alternative with a slightly reduced fluorescence level but increased homogeneity of fluorescence. Finally, the efficiency of engineered live biosensors was illustrated by the ultrahigh-throughput screening of *E. coli*-killing bacteria. *P. polymyxa* P4 strain producing potent anti-Gram-negative antibiotic polymyxin E was isolated using a single round of selection. The biosynthetic gene cluster (BGC) of polymyxin E has the same architecture as polymyxin A, D-Dab_3_-polymyxin B, and polymyxin E BGCs identified previously. The unique feature of the identified polymyxin E BGC is that its module 3 does not have an epimerization domain confirming the L-stereochemistry of Dab_3_ residue.

Engineered live biosensors provide a simple, efficient, and highly sensitive tool for precise monitoring of antimicrobial activity. Basic principles of their construction may be transferred to different Gram-negative bacteria. The implementation of ultrahigh-throughput technologies in antibiotic discovery enables deep profiling of antimicrobial activity, accelerating hit identification, and expanding biodiversity coverage. While a number of problems regarding the cultivation of unculturable microorganisms should be resolved to amplify the power of this technique, microfluidic droplet platforms already outperform classical cultivation strategies in some applications [26]. Another option is a search for specific potentiating agents targeting antibiotic resistance. In this case, using an AMR reporter strain supplemented with a conventional antimicrobial may be a target if the antibiotic-producing microorganism is resistant. It may be achieved using naturally resistant bacterial killers like fungi or engineered strains having improved efflux or mutation providing resistance. The application field of live biosensors is not restricted to microfluidics-based technologies like ultrahigh-throughput screening. Live biosensors may provide sensitive detection of antimicrobial activity in a broad sense, including such applications as routine antibiotic/probiotic screening, pollution monitoring, detection of antibiotic contamination, and more sophisticated fields based on synthetic biology principles.

## 4. Materials and Methods

**Genetic constructs and strains.** All genetic constructs were based on high-copy vector plasmid PURExpress Control DHFR Plasmid (NEB, Ipswich, MA, USA). Multiple cloning sites including *Xba*I, *Xho*I, and *Xma*I restriction sites flanked by *Nhe*I/*Hind*III sites were inserted to replace the T7 promoter-DHFR-T7 terminator region, resulting in pIvi-MCS vector. Promoter sequences were obtained by PCR assembly and cloned into pIvi-MCS digested with *Xba*I and *Xho*I. The pglpT is a strong constitutive promoter [18]. OXB20 is the strongest RecA promoter derivative with an ablated repressor binding site to enable constitutive expression (PSF-OXB20, OGS50, Sigma). The pJ23119 is the strongest promoter in a family of constitutive promoters isolated from a combinational library (PMID: 23560087). The pEm7 is a constitutive synthetic derivative of a T7 promoter (part Doulix biofundry, part ENW51Y). TagGFP2 and sfGFP genes were PCR amplified from pTagGFP2-N (Evrogen, Moscow, Russia) and pYTK047 plasmids, respectively, and cloned with *Xho*I/*Xma*I restriction sites. The pYTK047 was a gift from John Dueber (Addgene plasmid # 65154; http://n2t.net/addgene:65154; RRID:Addgene_65154, accessed on: 1 May 2018). The terminator region containing rrnB1 and T7 terminators was PCR amplified from pYTK047 and cloned with *Xma*I/*Hind*III restriction sites. The following *E. coli* strains were used: Rosetta (DE3) (Novagen, Madison, WI, USA), BL21(DE3) (Invitrogen, Waltham, MA, USA), TG1 (Lucigen, Middleton, WI, USA), XL-1 (Agilent, Santa Clara, CA, USA), and SHuffle T7 (NEB, Ipswich, MA, USA). Control GFP-producing reporter *S. aureus* cells were described previously [11,12,13].

**Fluorescence measurements.***E. coli* cultures were grown overnight using 2YT medium (16 g/L tryptone, 10 g/L yeast extract, 5 g/L NaCl) supplemented with 100 μg/mL ampicillin in shaking flasks at 37 °C and 250 rpm. Brain Heart Infusion (BHI) medium (BD, Franklin Lakes, NJ, USA) or TB medium (12 g/L tryptone, 24 g/L yeast extract, 4 g/L glycerol) was inoculated in 1:100 ratio and cultivated at 30 °C for 1, 2, and 4 days. Fluorescence measurements were made using Varioskan Flash Multimode plate reader (Thermo Fisher Scientific, Waltham, MA, USA) with λ_ex_/λ_em_ = 488/513 nm and NovoCyte Flow Cytometer (Agilent, Santa Clara, CA, USA). GFP-producing *E. coli* were visualized using an Eclipse Ti inverted fluorescence microscope (Nikon, Tokyo, Japan) with a standard FITC filter. Bacterial colonies grown on agar plates were analyzed by GFP fluorescence using VersaDoc (Bio-Rad, Hercules, CA, USA).

**Encapsulation of *E. coli* in droplets.** BL21(DE3) *E. coli*-producing sfGFP under control of pJ23119 promoter was cultured in BHI medium in shaking flasks at 37 °C and 250 rpm until early logarithmic growth phase. Subsequently, liquid cultures were filtered using 40-μm cell strainers (Greiner Bio-One) and 20-µm solvent filters (A-313, IDEX, Northbrook, IL, USA) and then diluted to reach OD_600_ = 0.3 (occupancy (λ) ~ 5 *E. coli* cells per a droplet). *E. coli* cells were encapsulated in droplets of microfluidic double emulsion (MDE), using 20-µm microfluidic chips produced via soft lithography, as was described previously [11]. MDE droplets with encapsulated bacterial cells were cultured at 30 °C in a water vapor saturated incubator. MDE droplets were loaded into a hemocytometer and were visualized using an Eclipse Ti inverted fluorescence microscope (Nikon) with a standard FITC filter.

**Ultrahigh-throughput screening of anti-Gram-negative antibiotic activity.** The selection of bacteria displaying antibacterial activity was described in detail previously [11,12,13]. Briefly, sfGFP-producing *E. coli* cells were vitally stained with sulfo-Cyanine5 NHS (Lumiprobe, Moscow, Russia), washed, and filtered using 20-µm solvent filters (A-313, IDEX, Northbrook, IL, USA). The soil microbiome was isolated in the Moscow region. Microbiome samples were unfrozen directly before encapsulation, resuspended in BHI broth (BD, Franklin Lakes, NJ, USA), and filtered through 40-µm cell strainers (Greiner Bio-One, Monroe, NC, USA). The sfGFP-producing live biosensors were co-encapsulated with a soil microbiome suspension in droplets of MDE. After overnight incubation at 35 °C, Calcein Violet AM (Thermo Fisher Scientific, Waltham, MA, USA) was added to the droplet emulsion to the final concentration of 10 µM. Subsequently, the droplets with simultaneous sCy5^high^, GFP^low^, and Calcein Violet^high^ fluorescence were sorted using a FACSAria III cell sorter (BD, Franklin Lakes, NJ, USA). Bacterial colonies were regenerated after plating on BHI–agar (BD, Franklin Lakes, NJ, USA) and tested for antibiotic activity against *E. coli* using the agar overlay assay.

***P. polymyxa* cultivation.***P. polymyxa* P4 was cultivated in SYC medium containing 40 g/L sucrose, 5 g/L yeast extract, 4 g/L CaCO_3_, 1.5 g/L K_2_HPO_4_, 2 g/L glucose, 2 g/L NaCl, 1.5 g/L MgSO_4_, 2 g/L (NH_4_)_2_SO_4_, 0.01 g/L FeSO_4_, and 0.01 g/L MnCl_2_ for 24 h at 30 °C. *P. polymyxa* P4 was inoculated from overnight culture using 1:100 dilution and cultivated using 750-mL flasks in 100 mL with 250 rpm shaking for 4 days.

**Antimicrobial activity**. Inhibition of bacterial cell growth was measured by a doubling dilution of *P. polymyxa* P4 medium and C18 HPLC fractions in 2YT medium inoculated with *E. coli* OD_600_ = 0.002. After overnight incubation at 30 °C, *E. coli* growth was analyzed by GFP fluorescence (λ_ex_/λ_em_ = 488/513 nm) and OD_600_ using a Varioskan Flash Multimode Reader (Thermo Fisher Scientific).

**Whole-genome sequencing and bioinformatic analysis.** Total DNA was isolated using the QIAamp DNA Investigator Kit (Qiagen, Germantown, MD, USA). Genomic DNA was disrupted into 400–550-bp fragments by Covaris S220 System (Covaris, Woburn, MA, USA). Fragment libraries were prepared using the NEBNext^®^ DNA Library Prep Reagent Set for Illumina and the NEBNext^®^ Multiplex Oligos for Illumina^®^ (96 Index Primers) (Illumina, San Diego, CA, USA) according to the manufacturer’s instructions. Sequencing of libraries was performed using the genetic analyzer HiSeq2500, the HiSeq^®^ PE Cluster Kit v4–cBot™, and the HiSeq^®^ SBS Kit v4 (250 cycles) (Illumina, San Diego, CA, USA) according to the manufacturer’s instructions. Genome assemblies were performed using SPAdes 3.9.0 [29]. Genomes were annotated with prokka [30]. Identification of biosynthetic gene clusters and NRPS modular organization was performed with antiSMASH 6.0 [31]. Comparative analysis of homologous gene clusters was provided by MultiGeneBlast [32] based on the MIBiG database [33].

**Active metabolite extraction and metabolomic analysis.** The culture medium of *P. polymyxa* P4 was centrifuged at 10,000× *g* for 10 min. Active metabolites were extracted by solid-phase extraction with LPS-500 sorbent (Technosorbent, Moscow, Russia) using buffer A (10 mM NH_4_OAc pH 6.0, 20% ACN) for sorbent wash and buffer B (10 mM NH_4_OAc pH 6.0, 80% ACN) for elution. LC-MS analysis was carried out on an Ultimate 3000 RSLCnano HPLC system connected to an Orbitrap Fusion Lumos mass spectrometer (ThermoFisher Scientific) with the loading pump used for analytical flow gradient delivery. Samples were separated on Luna Omega C18 1.6 µm 100 Å column 100 × 2.1 mm at a 200 µL/min flow rate. Separation was done by a gradient of 99.9% ACN, 10 mM ammonium formate, 0.1% FA (Buffer B) in 99.9% H_2_O, 10 mM ammonium formate, 0.1% FA (Buffer A): 5% B at 0 min, 5% B at 5 min, 99% B at 20 min, followed by 5 min wash at 99% B and 10 min equilibration at 5% B before the next run. UV data were collected at 260 and 315 nm. MS1 spectra were collected in Positive ion mode at 30 K Orbitrap resolution in profile mode with 200–2000 a.e.m mass range and RF lens 30%. For the rest of the MS1 parameters as well as for the ESI parameters, the default values suggested by Xcalibur software ver. 4.3.73.11 were taken. MS2 precursors were selected based on MS1 intensity: Intensity threshold was 5 × 10^4^ with the dynamic exclusion set to 10 s after two selections with the mass tolerance of 10 ppm and isotope exclusion. MS2 spectra were collected at 15 K resolution in centroid mode. The isolation window was set to 1/6 m/z with no offset and Quadrupole isolation mode. Fragmentation was done by HCD with a stepped CE of 20, 35, and 50%. The rest of the MS2 parameters were taken as default values. The total MS1-MS2 cycle time was selected to 1 sec. LC-MS/MS raw data were analyzed in Compound Discoverer 3.2 (Thermo Fisher Scientific). Peak annotation was performed with ChemSpider, Natural Product Atlas 2020, and MzCloud databases using MS1 and MS1-MS2 information correspondingly with 5 ppm mass accuracy, isotopic distribution ≥ 50%, and match score ≥ 85%.

## Data Availability

Data is contained within the article. Additional data are freely available on request from the corresponding author.
